# iTRAQ-Based Quantitative Proteomic Analysis on S100 Calcium Binding Protein A2 in Metastasis of Laryngeal Cancer

**DOI:** 10.1371/journal.pone.0122322

**Published:** 2015-04-13

**Authors:** Cong Zha, Xue Hua Jiang, Shi Fang Peng

**Affiliations:** 1 West China School of Pharmacy, Sichuan University, Chengdu, Sichuan, China; 2 Department of Infectious Diseases, Xiangya Hospital, Central South University, Changsha, Hunan, China; 3 Department of Health Management Center, Xiangya Hospital, Central South University, Changsha, Hunan, China; Instituto Tecnologia Quimica e Biologica; Universidade Nova de Lisboa, PORTUGAL

## Abstract

Laryngeal cancer is the most frequent neoplasm in the head and neck region, with the vast majority of tumors originating from squamous cells. The survival rate of patients with laryngeal cancer has not improved substantially over the past 25 years. To acquire further knowledge regarding the molecules responsible for laryngeal cancer oncogenesis and, in turn, to improve target therapy，iTRAQ and mass spectrometry analysis were utilized to detect differences in protein expression from 15 paired laryngeal cancer and adjacent non-cancerous tissue samples. Using mass spectrometry analysis, the expression levels of 100 proteins in laryngeal cancer samples were distinct from the non-tumor, non-cancerous samples. Further validation of the differentially expressed proteins S100A2, KRT16, FGB and HSPB1 were carried out using quantitative real-time RT-PCR, immunoblot and immunohistochemistry. Functional analysis of one of the highly expressed proteins, S100 calcium binding protein A2 (S100A2), was performed using RNA interference. As a consequence, attenuated S100A2 expression enhanced the ability of HEp-2 cell lines to migrate and invade *in vitro*. Our investigation complements the current understanding of laryngeal cancer progression. Furthermore, this study supports the concept that enhanced expression of S100A2 may be a promising strategy in developing novel cancer therapeutic drugs.

## Introduction

Laryngeal carcinoma is the most common type of cancer in the head and neck, and accounts for 2.4% of new malignancies worldwide each year [1,2]. Since there is a lack of reliable and early diagnostic biomarkers, the disease is often diagnosed at an advanced stage, thereby missing important opportunities for treatment. The outcome often results in the loss of laryngeal function. It is known that laryngeal carcinoma may spread, by direct extension, to adjacent structures, frequently as distant metastases of the lungs [3, 4]. Clinical therapies of laryngeal carcinoma mainly include surgery and chemoradiotherapy. Deprivation of the ability to speak or swallow, and local failure and/or late complications as a result of the carcinoma are sources of frustration for patients as well as the medical community which services them. Moreover, the survival rate of patients with laryngeal cancer has not improved substantially in the last few decades [5]. Considering the diminished living conditions, poor survival rate, and the importance of the larynx in daily or social functions, it is urgent to provide further insight into the mechanism of laryngeal carcinoma, with the expectation of finding more effective and alternative molecular targets for predicting this disease.

Proteomics is considered to be a powerful tool for global evaluation of protein expression. It has been suggested that analysis of the cancer proteome can be beneficial to understand not only the association between protein alterations and malignancy, but also the effect of molecular intracellular mislocalization in tumor initiation [6]. Li et al. utilized 2D-LC-MS/MS to identify 141 up-regulated proteins and 140 down-regulated proteins in laryngeal carcinomas and demonstrated PFN1 was strongly associated with the ability of cancer cells to proliferate and migrate [7]. Moreover, using 2-DE MALDI-TOF analysis, Bijon Chatterji et al. identified disease regulated serum proteins in the lung cancer of c-myc transgenic mice, suggesting that the serum amyloid P component was uniquely expressed in the late stages of cancer [8]. Nevertheless, proteomics study has been limited due to the intrinsic disadvantages of 2-DE, such as inter-gel variation, low sensitivity and excessive time/labor cost [9]. Recently, isotope-based quantitative proteomics has made breakthroughs in various fields of research. Owing to its ultrasensitive and high-throughput property, isobaric tags for relative and absolute quantitation (iTRAQ) coupled with 2-dimensional liquid chromatographyn and tandem mass spectrometry (MS/MS) analysis, it has been deemed to be one of the most reliable methods of analysis.

On account of lacking clinically established biomarkers available for early detection and therapeutic targets of laryngeal carcinoma, we utilized the iTRAQ method to analyze differentially expressed proteins between laryngeal carcinoma and non-tumor, non-cancerous counterparts, identifying indicated molecules to assist in diagnosis and/or to be used as therapeutic targets.

## Materials and Methods

### Tissues and Cell Line

The approval of the Institutional Review Board for Human Subject Review was obtained before this investigation was performed. This project was approved by the Medical Ethics Committee of Chongqing Medical University, China. All human participants provided written informed consent and all clinical investigations were conducted according to the principles expressed in the Declaration of Helsinki [10]. Two specimens, laryngeal carcinoma tissues and the corresponding adjacent noncancerous tissues were obtained from each patient and then examined by experienced pathologists. In total, fifteen laryngeal carcinoma samples and fifteen paired non-cancerous samples were collected from 15 patients who underwent surgical resection in the Second Affiliated Hospital of Chongqing Medical University. The clinical details of the patients are shown in S1 Table. The human laryngeal carcinoma cell line HEp-2 was purchased from the cell bank of the Shanghai Institute of Cell Biology (Shanghai, China).

### Protein preparation

Total proteins were extracted from fifteen laryngeal carcinoma samples and fifteen non-tumor benign samples. 100μg of total proteins from each sample were denatured, cysteine blocked, and digested with trypsin as described in the standard protocol of iTRAQ (AB SCIEX, USA). Pooled non-tumor samples were labeled with iTRAQ tags 117 and 119, and tags 118 and 121 were used to label pooled laryngeal carcinoma samples. The labeled peptides were pooled in 1:1:1:1 ratios and lyophilized. The mixed peptides were dissolved in mobile phase A (20 mM ammonium formate, pH 10.0), separated with an LC-30A high performance liquid chromatography system (Shimadzu, Japan) and eluted on the Gemini- NX C18 column (4.6mm × 250mm, 5μm 110Å, Phenomenex, USA) at a flow rate of 800μl/min with a gradient of 5–23% mobile phase B (80% v/v acetonitrile, 20mM ammonium formate, pH 10.0) over 20 min, 23–45% B over 15 min and, finally, by ramping up to 90% B in 1 min and holding for 4 min. The absorbance at 220 nm was monitored. A total of 10 fractions were collected and lyophilized for further analysis.

### NanoLC-TripleTOF 5600analysis

A TripleTOF 5600 system coupled with an Eksigent NanoLC- 2D system (AB Sciex) was used for protein identification and quantization. Peptide fractions obtained from labeling and fractionation were reconstituted in 30μl mobile phase A (2% acetonitrile with 0.1% formic acid), and loaded on a C18 trap column (5 μm, 0.3 mm × 5 mm, Agilent). After being desalted at a flow rate of 2μl/min for 10 min, samples were separated on a house-packed nanoLC C18 column (200 Å, 5μm, 75μm × 10 cm) with the elution gradient of 8–25% mobile phase B (98% acetonitrile with 0.1% formic acid) over 85 min, 25–50% B over 20 min and, finally, ramping up to 80% B in 1 min and holding for 4 min at a flow rate of 300nl/min. The TOFMS scan was set in the positive ion mode at a mass range of 350-1500m/z with a 0.25 sec accumulation time, followed by information-dependent acquisition (IDA). The top 30 precursor ions within each cycle were automatically selected for fragmentation with each MS/MS spectrum accumulated for 0.1 sec (100-1500m/z).

### Data analysis

ProteinPilot v.4.5 software (AB Sciex) was used for data searching against the UniProt database. A standard parameter set was used for the search, which included Homo sapiens, iTRAQ reagent 8 plex, Cysteine modification by methylmethanethiosulfonate, trypsin digestion, TripleTOF 5600system, thorough ID, biological modifications, 0.05 (10.0%) detected protein threshold and false discovery rate analysis. A threshold of confidence above 95% and a local false discovery rate of less than 1% were used for both protein identification and quantitative analysis. More than two unique peptides were required for protein identification. P-values<0.05 were required for relative quantification. PeakView 1.1 software was used to extract ion chromatograms.

### Quantitative Real Time-PCR Analysis

Tissues were treated with TRIzol (Gibco BRL, MD) and RNA was extracted and reverse-transcribed as previously described [11]. The experiments were performed according to the manufacturer’s instructions. Quantitative RT-PCR assays were performed on an ABI 7900HT system with TaqMan Kits. Primers for POSTN (HP209418), KRT17 (HP200402), MPO (HP200232), S100A2 (HP209010), FGB (HP208334), HSPB1 (HP205401), FN1 (HP234005), AGR2 (HP209366), ORM1 (HP200574), ALB (HP200449), A2M (HP200001), KRT4 (HP206000) and GAPDH (HP205798) were used. The 2^- ΔΔCT^ method was performed to analyze the relative changes in gene expression [12]. Similar results were obtained when experiments were performed in triplicate.

### Western Blotting Analysis

Western Blotting analyses were done as previously described [11]. Briefly, after lysing using a non-ionic detergent (NIDs) buffer [13], the concentration of tissues/cells lysates were determined. The samples (20μg protein) were subjected to SDS–PAGE followed by transfer to a polyvinylpyrrolidone membrane (Amersham Biosciences, Sweden). After incubating with primary antibodies (1:500–1:1000) against S100A2, POSTN, FN1, KRT17 and FGB (Abcam, USA) and washing with TBS-T, HRP-conjugated (HRP) secondary antibodies (1:5000, Amersham Biosciences) were used. The bands were detected with an ECL detection system (Amersham Biosciences) [11]. The experiments were performed in triplicate.

### Immunohistochemistry (IHC) and Tissue Microarrays(TMA)

The tissue microarrays (LP804) were purchased from U.S. Biomax, Inc. to detect POSTN, S100A2, KRT17, and FN1 in cores from 60 laryngeal carcinoma tissues and 20 non-tumor tissues. Graded ethanol was used to deparaffinize the cores. H_2_O_2_ was used to quench the endogenous peroxidase activity. After blocking, antibodies against S100A2 (1: 90), KRT17 (1: 200), POSTN (1:150), and FN1 (1: 200) were added and incubated at 4°C overnight. Detection was performed with the Envision/horseradish peroxidase system (Dako-Cytomation, Denmark) [13].

Semi-quantification of protein expression was defined by scoring criteria as previously described [14]. The positive cells (%) and staining intensity (scale 0–3) were checked, which were then multiplied to yield a score from 0 to 300. In order to maintain consistency, the same qualified pathologist gave interpretations for all IHC data.

### Functional Assays

HEp-2 cells were trypsinized and counted. 50 nM of S100A2-targeted siRNAs or control siRNA was transfected into HEp-2 cells (10^5^ cells/well). After 48 hrs, wound healing and invasion experiments were performed on 6-well plates seeded with HEp-2 cells as described previously [14]. After the cells reached confluence, a 200μl pipette tip was used to incise the cell monolayer. The debris was then rinsed away and removed. The extent of gap closure was monitored and photographed under a microscope for up to 24 hours. The invasion assays were performed using a Cell Invasion Assay Kit (Cell Biolabs) according to the manufacturer’s instructions. After 24 hours, the number of cells that invaded and attached to the bottom of chamber was measured by CyQuant GR fluorescent dye (560 nm).

### Statistics

Statistical analyses were performed with SPSS software v13.0 using Student-t test, Mann-Whitney U-test, χ2 test and Spearman’s rank correlation analysis. A P-value of less than 0.05 was considered statistically significant. All tests of significance were two-tailed.

## Results

### Differentially Expressed Proteins in Laryngeal Carcinoma

**Fig 1:**
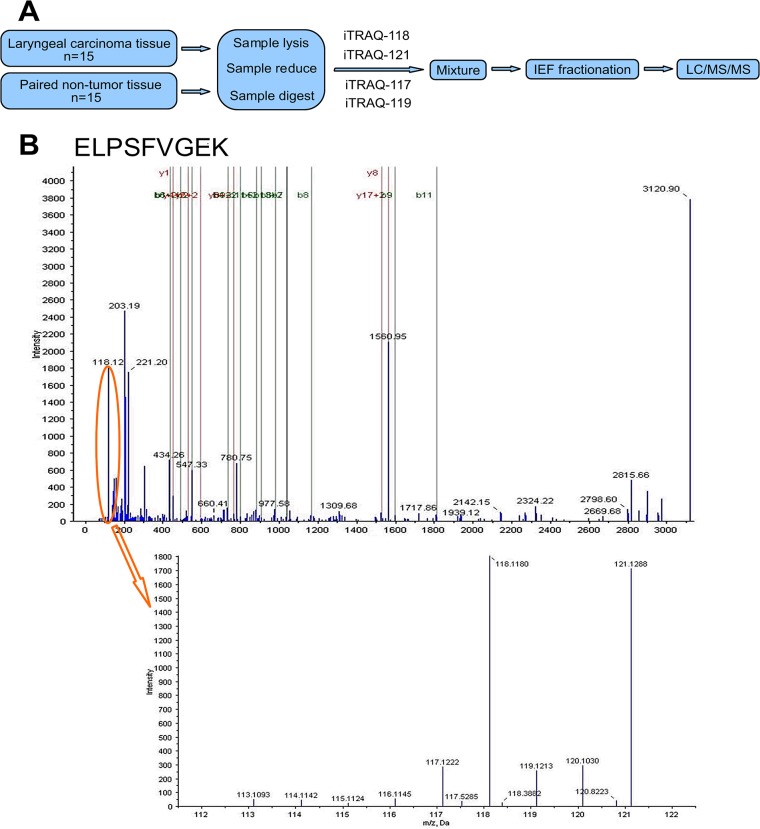
(a) Schematic Flowchart of the iTRAQ method. (b) Relative abundance of S100A2.

The schematic flowchart of the iTRAQ method is shown in Fig 1. iTRAQ 117 and 119 tags were used to label the fifteen pooled non-tumor samples and iTRAQ 118 and 121 tags were used for the fifteen pooled cancerous tumor samples. Thus, the ratio of 118:117 and 121:119 indicates the relative abundance of S100A2 protein (Fig 1B) in pooled cancerous tumor tissues compared to that of the control group.

As described previously, the protein identification threshold, a ProtScore of more than 1.3, was used to obtain a confidence level of 95%. For the subsequent relative quantification analysis, despite statistical analysis being part of the ProteinPilot software, an additional > 1.3 or < 0.77 (1/1.3)-fold cutoff was applied to all iTRAQ ratios to minimize false positives when identifying proteins as over- or under-expressed. This cutoff value was adopted since overall technical variation of data from the duplicate experiments was estimated to be below 30%, and in other investigations using the iTRAQ approach, this value is widely employed [15–18]. Thus, the lower and upper limits were 0.77 (1/1.3) and 1.3 (1×1.3), respectively [11]. Proteins with iTRAQ ratios below 0.77 were considered to be under-expressed, while those whose ratios which were more than 1.3 were deemed to be over expressed [11]. Employing this strategy, when compared against non-tumor tissues, 51 up-regulated proteins and 49 down-regulated proteins in pooled tumor tissues were found (S2 Table).

### Classification of Differentially Expressed Proteins

**Fig 2:**
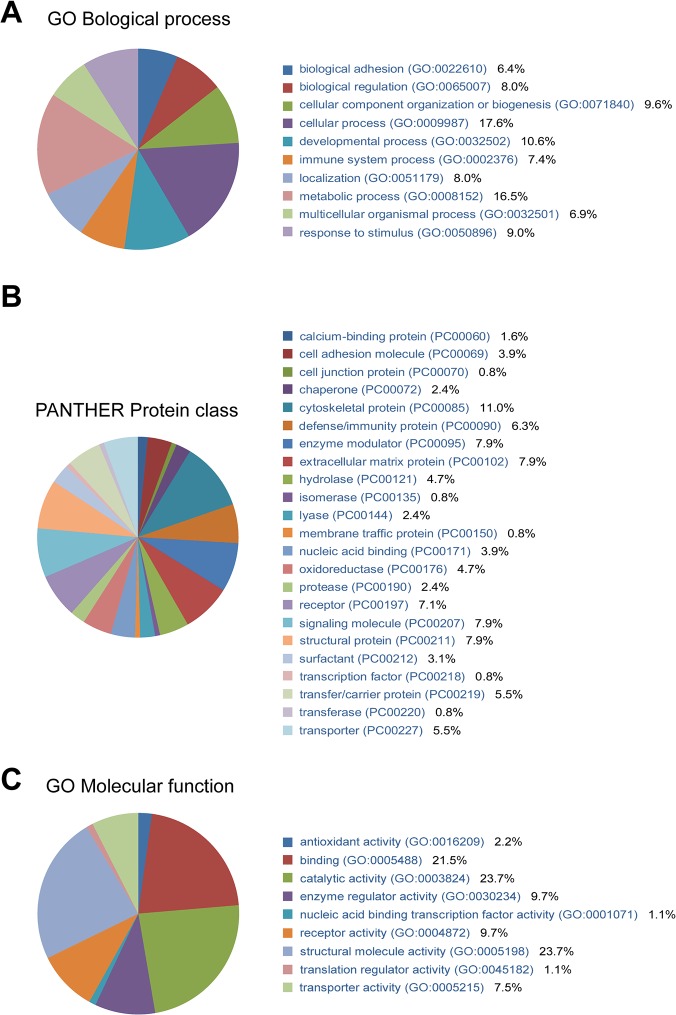
Differentially expressed proteins categorized by (a) biological process, (b) PANTHER protein class and (c) molecular function.

We classified 100 differentially expressed proteins associated with laryngeal carcinoma using PANTHER (Protein Analysis through Evolutionary Relationships) Classification System (www.pantherdb.org) to obtain a better understanding of their molecular and functional characteristics. In total, 10 biological processes, 23 protein classes, and 9 molecular functions (Fig 2) were classified. The top three biological process categories were cellular (17.6%), metabolic (16.5%) and developmental processes (10.6%) (Fig 2A). The top three protein class categories were cytoskeletal protein (11%), enzyme modulator (7.9%) and signaling molecule (7.9%) (Fig 2B). The top three molecular function categories were catalytic (23.7%), structural molecule (23.7%) and binding activities (21.5%) (Fig 2C). Details are shown in S3, S4 and S5 Tables.

### Validation of Differentially Expressed Proteins

**Fig 3:**
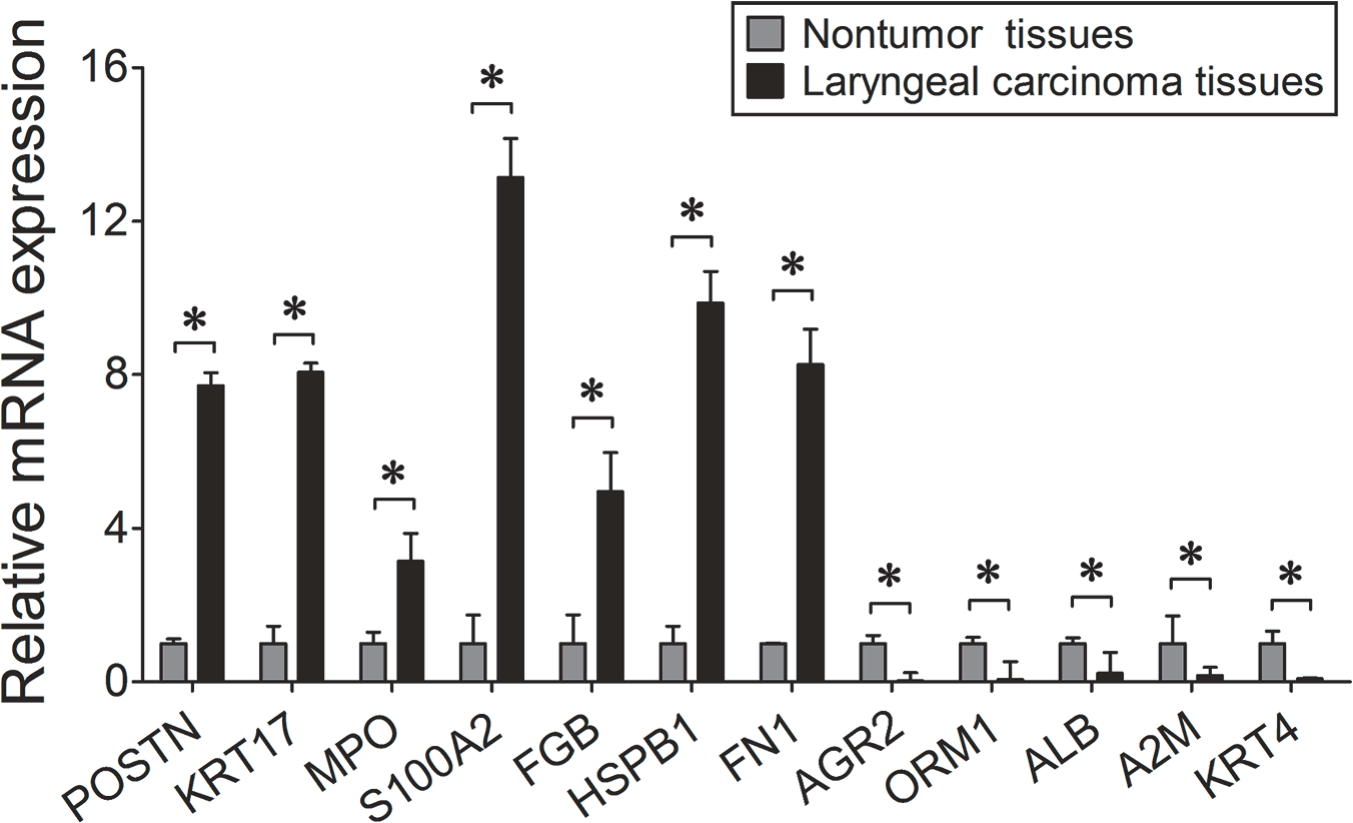
Relative expression levels of 12 mRNAs, normalized to GAPDH.

**Fig 4:**
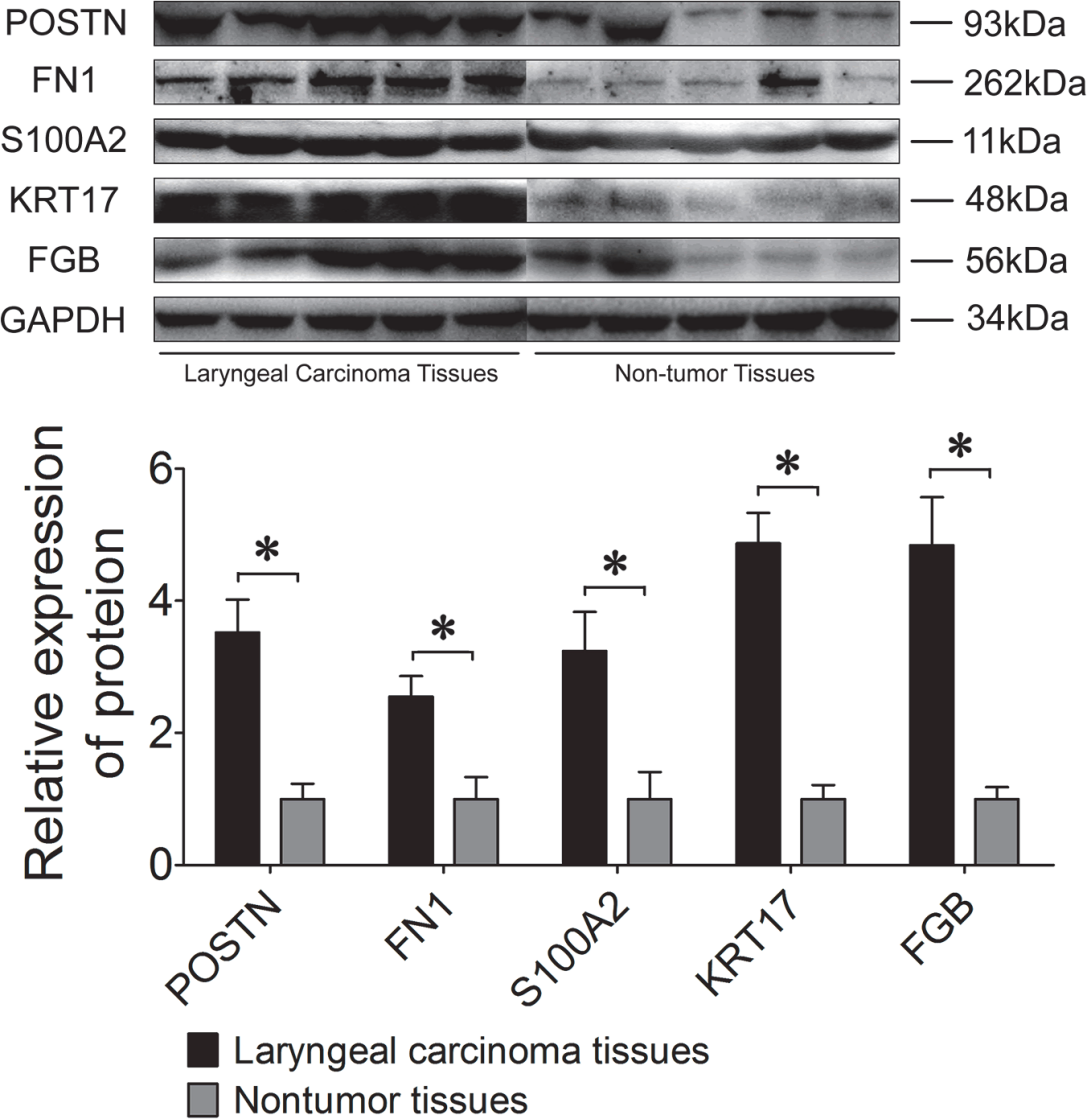
A representative Western Blot for expression of S100A2, FGB, KRT17, FN1 and POSTN in the laryngeal carcinoma tissues.

Several differentially expressed proteins were chosen for further validation according to their most significant ratios or their relevance to carcinoma disease. Fig 3 shows the relative mRNA expression levels of POSTN, KRT17, MPO, S100A2, FGB, HSPB1, FN1, AGR2, ORM1, ALB, A2M, KRT4, as normalized to GADPH. Among them, the levels of POSTN, KRT17, MPO, S100A2, FGB, HSPB1, and FN1 were found to be up-regulated in the laryngeal carcinoma tissues, whereas the rest were down-regulated, compared to the normal tissues. Fig 4 shows expression of S100A2, FGB, KRT17, FN1 and POSTN in the laryngeal carcinoma tissues detected using Western blot analysis. It noted up-regulation of S100A2, FGB, KRT17, FN1 and POSTN in laryngeal carcinoma tissues, as compared with normal tissue. This trend matched with what had been previously observed using the iTRAQ method.

### Validation of S100A2, POSTN, KRT17 and FN1 Using Tissue Microarrays

**Fig 5:**
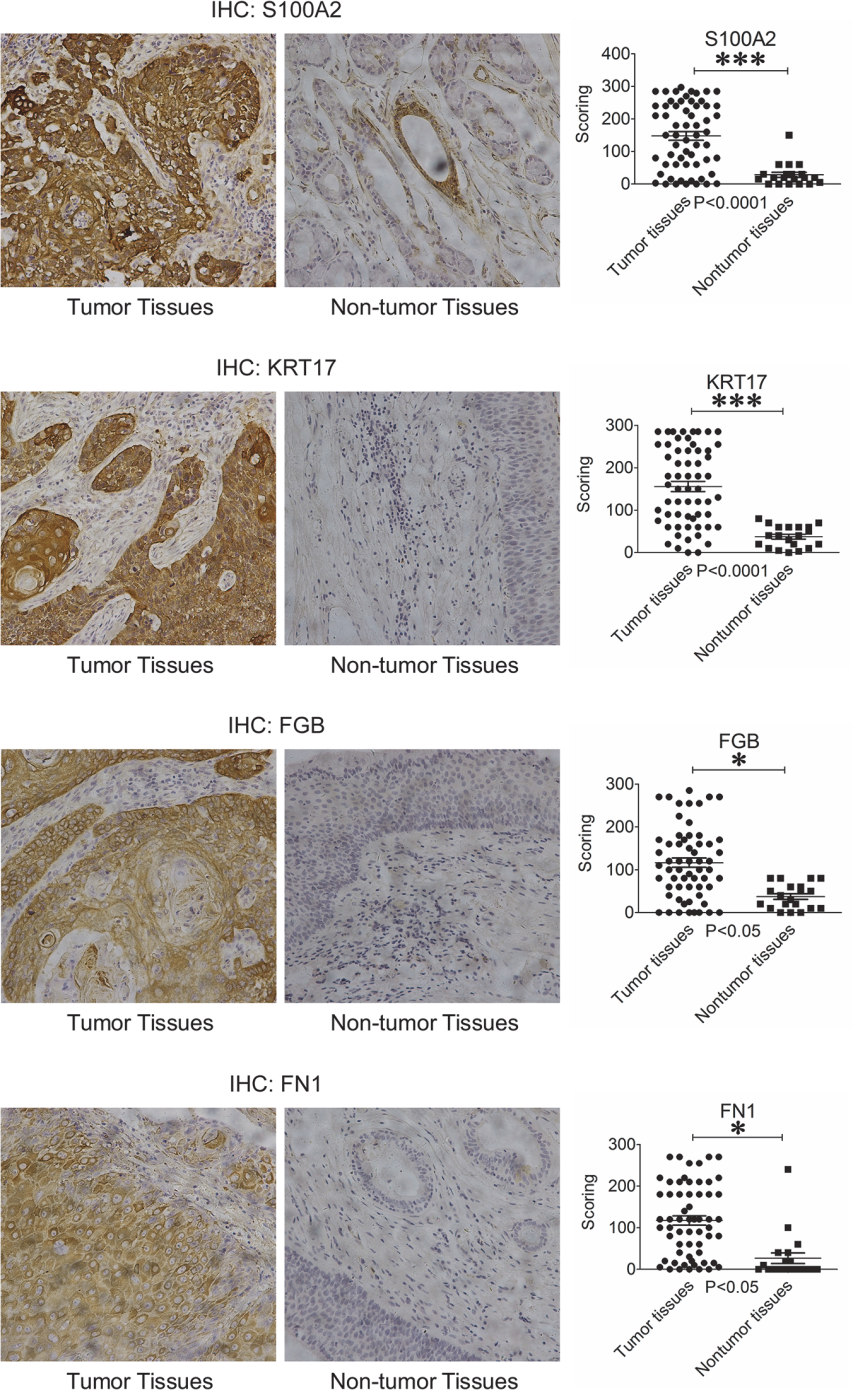
Representative immunohistochemistry images of S100A2, FN1, KRT17 and POSTN in tumor (left) and non-tumor (right) tissue samples.

We performed IHC to assess the clinical relevance of S100A2, FN1, KRT17 and POSTN. An array including 60 laryngeal carcinoma tissues and 20 matched or unmatched tumor adjacent tissues were used. As shown in Fig 5, a significant number of laryngeal carcinoma tissues had higher levels of S100A2 than in matched or unmatched non-tumor adjacent tissues. Specifically, 55/60 (92%) of laryngeal carcinoma samples expressed S100A2, as opposed to 15/20 (75%) in non-tumor samples. Moreover, the S100A2 staining intensity in IHC was stronger in the laryngeal cancer samples.

### Roles of S100A2 in Laryngeal Carcinoma Cells

**Fig 6:**
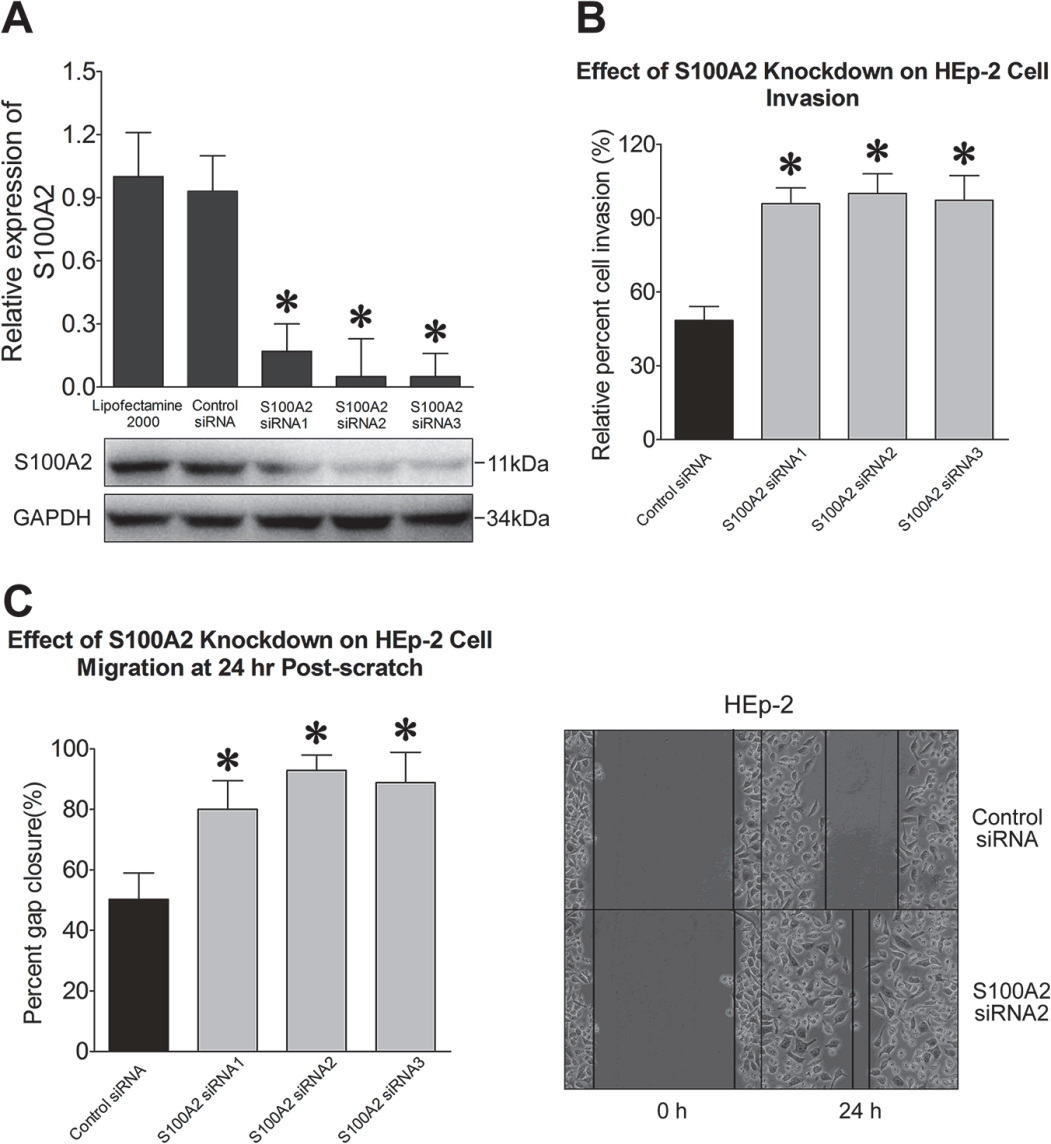
Representative images of functional studies of HEp-2 cells. (a) Relative expression of S100A2 in siRNA silenced cells. (b) Cell invasion capacity of S100A2-silenced HEp-2 cells. (c) Cell migration capability in S100A2-silenced HEp-2 cells.

The dramatic increase of S100A2 in laryngeal carcinoma tissues suggests that S100A2 is involved in metabolic processes that are not only involved in the biosynthesis of laryngeal carcinoma cells, but also are crucial for the carcinoma cells abilities to migrate and invade. To test this hypothesis, a laryngeal carcinoma cell line Hep-2 was tested with an RNA interference assay. As expected, the S100A2-specific sequences silenced the S100A2 expression (Fig 6A). Control and S100A2-silenced cells were then subjected to invasion and migration assays. Invasion capacity of S100A2-silenced cells was enhanced by 35–42% when compared to the control group (p<0.01) (Fig 6B). Similarly, the readout of the scratch wound repair assays was increased by 36–47% in S100A2-silenced Hep-2 cells when compared to control cells (p<0.01), due to S100A2 knockdown (Fig 6C). Our results supported the concept that S100A2 may be an effective target in HNSCC treatment.

## Discussion

Laryngeal cancer is the most frequent neoplasm in the head and neck region. Typically, head and neck squamous cell carcinoma (HNSCC) cells show persistent invasion that frequently leads to local recurrence and distant lymphatic metastasis. However, molecular mechanisms associated with the invasion and metastasis of HNSCC has remained poorly understood. Due to the limited understanding of the mechanism behind laryngeal cancer development, the survival rate of patients with laryngeal cancer has not improved substantially over the past 25 years [19]. Therefore, it is urgent that the relevant mechanism to complete our understanding of laryngeal cancer be explored.

Because of the low resolution, the differentially expressed proteins detected in laryngeal carcinoma tissue using 2D-MS were limited to some high-abundance proteins [20]. In the proteomics study of Li et al. [21] using two-dimensional strong cation-exchange/reversed-phase nano-scale liquid chromatography/mass spectrometry analysis, 281 differential proteins in 34 laryngeal carcinoma tissues were identified. Among them, 23 candidate proteins were found to be comparable with our data while 9 proteins presented a reversed expression trend. The amount of identified proteins and the discrepancies might be due to the range of cut-off values defining differential proteins and the varying ability of mass spectrometers to identify a particular protein. Hence, reliable proteomic technologies and multi-verification processes are critical to the discovery of disease-specific biomarkers. Additionally, in more than one proteomics study [20,22], cystatin-B, heat shock protein beta-1, and isoform 1 of serum albumin were deemed to be related to laryngeal carcinoma, but without in-depth functional research or clinical validation. In our investigation, we identified proteins differentially expressed between tumor and non-tumor laryngeal carcinoma via the iTRAQ proteomic approach. Verification studies using qRT-PCR, immunoblot and IHC assays confirmed that the expressions of S100A2, POSTN, FN1 and KRT17 were, indeed, significantly increased in tumor tissues. Functional studies indicated that S100A2 tends to affect the ability of laryngeal cancer cells to migrate and invade. Our findings revealed that the iTRAQ method for large-scale protein quantification was amenable to high throughput and was able to provide credible results, and some novel proteins uncovered here may serve as potential targets for laryngeal carcinoma treatment.

S100A2, previously labeled S100L or CaN19, encodes a 99-amino acid protein that is a member of the calcium-binding proteins of the EF-hand family[23]. Although it has played an adverse role in different kinds of cancers, there has been growing interest in it as a tumor-suppressor in other types of cancers. In the research of Nagy et al., they showed that S100A2 had a clear inhibitory influence on cell motility in the case of human head and neck squamous cell carcinoma lines [24]. Tsai et al. demonstrated that ectopic overexpression of S100A2 induced G1/S cell cycle arrest, thus attenuating cell growth both in vitro and in vivo partially by down-regulation of Cox-2 in oral cancer cells [25]. Feng et al. found that *S100A2* gene played a potential role as a tumor suppressor in lung carcinogenesis, which may result from its DNA methylation status in the promoter region of the *S100A2* gene [26]. Taken together, in this context, a tumor-suppressor role for S100A2 is proposed. Since uncontrolled cell growth and invasion/metastasis are two characteristic features of cancer, we investigated whether S100A2 could play a tumor suppressor role in certain laryngeal cancer cell lines by inhibiting cell invasion and migration. In accordance with this hypothesis, our results interpreted that invasion or migration capacity of HEp-2 cells were enhanced by ~40% or ~45%, by down-regulating the expression of S100A2 protein through S100A2-specific RNA interference experimentation. In line with the observed results in the HNSCC cell line RPMI, which showed a relatively low level of S100A2 expression, external addition of S100A2 strongly enhanced the cell migration capacity. This effect has been confirmed in another HNSCC cell line FADU, which highly expresses S100A2, where antisense oligonucleotide treatment can stimulate cell motility [27]. Similarly, by using squamous cell carcinoma (SCC) as a cancer model, it was found that overexpressed S100A2 possesses a profound effect in inhibiting cell migration and invasion in vitro and in vivo [25]. Tan et al. revealed that the S100A2 promoter was transcriptionally activated by wild-type p53 in a dose-dependent as well as a p53 binding site-dependent manner. The p53-induced transactivation of the S100A2 promoter was enhanced by etoposide, a p53 activator, and blocked by a dominant negative p53 mutant [28]. Intriguingly, S100A2 could interact with p53 in a calcium-dependent manner and activate p53 transcriptional activity, which presumably helps restore p53 function in cell arrest and apoptosis [29]. The potential effects of p53 in tumor metastasis [30,31] and its interplay with S100A2 could in turn explain the function of S100A2 in suppressing the invasion and migration capacity of HEp-2 cells in vitro. In line with the suggestion that S100A2 be considered as a tumor-suppressor candidate, our study suggests that S100A2 may prevent laryngeal cancer cells from invading and migrating in vitro via the p53 signaling pathway. As the overexpression of S100A2 was detected in laryngeal cancer and siRNA gene knockdown abrogates cell invasion and migration, S100A2 could be also developed into a biomarker of malignancy in laryngeal cancer. In the immunohistochemical analysis of 62 untreated laryngeal carcinoma patients [32], Libero Lauriola noted a higher level of S100A2 was associated with well-differentiated tumors and less lymph node involvement. Another study on samples of gastric adenocarcinoma [33] pointed out that loss of S100A2 expression was significantly associated with lymph node metastasis, lymphatic vessel invasion, and depth of invasion. These results indicated that patients with low grade S100A2 should undergo an aggressive initial treatment and a much less aggressive treatment should be suggested for patients with high grade S100A2.

Another protein found in our study to be remarkably up-regulated in laryngocarcinoma tissues was Keratin 17 (KRT17), which is a component of cytoskeleton protein. The main function of Keratin 17 is in the formation and maintenance of various skin appendages [34], and is specifically considered to be a marker of basal cell differentiation in complex epithelia [35]. It was reported that KRT17 could be a tumor marker in squamous cell carcinoma of head and neck and breast cancers [36,37]. Since increased KRT17 could activate and bind progesterone receptors (PR) and HER2, KRT17 overexpression was associated with a high tumor grade and positive axillary lymph nodes [38,39]. Besides, Keratin 17 could promote epithelial proliferation and tumor growth by polarizing the immune response in skin mucosa [40]. Another study noted that down-regulation of Keratin 17 expression could inhibit the proliferation of T cells and the production of interferon γ(IFN-γ) effectively in psoriatic cases, which means that Keratin 17 could be used as a new target for the treatment of psoriasis [41]. In our study, we demonstrated increased KRT17 expression in laryngocarcinoma tissues compared to non-tumor tissues by proteomic profiling, and confirmed this trend by western blot and RT-PCR. However, the relevance of KRT17 and tumor progression in laryngocarcinoma remains elusive, and the mechanisms involved still require further investigation.

Fibronectin 1 (FN1), which was found up-regulated in laryngeal cancer tissues in our study, is a glycoprotein that is involved in cellular adhesion and migration processes including wound healing, blood coagulation, host defense, and metastasis [42]. Overexpression of FN1 protein was found in hepatocellular carcinoma [43], gastrointestinal cancer and head/neck cancer [44]. In the previous study of Aye Thant et al., they showed that treatment of NOM1 ovarian cancer cells with fibronectin 1 (FN1) stimulated matrix metalloproteinase (MMP)-9 secretion activated the invasiveness of cells via the FAK/Ras signaling pathway [45,46]. They also reported that PI-3 kinase inhibitors, wortmannin and LY294002, dramatically suppressed the FN-dependent secretion of MMP-9 and inhibited Akt activation, which ultimately suppressed the invasiveness of NOM1 ovarian cancer cells [47]. In addition, S Jagadeeshan et al. illustrated that Pak1–NF-kB–p65-mediated FN1 regulation is a potent tumor-promoting mechanism in pancreatic cancer cell mode without KRAS mutations [48]. In the case of down-regulated fibronectin via short hairpin RNA–mediated silencing of gene expression, the proliferation of brain tumors was impaired, which was due to inhibition of the Src kinase–dependent survivin activity [49]. Combining this information and the overexpression of FN1 in our study, it is assumed that FN1 plays a role in the development of laryngeal cancer. Further investigations to reveal this mechanism is to be encouraged.

Periostin (POSTN), also called osteoblast-specific factor 2, belongs to the superfamily of TGF-β-inducible proteins [50]. Previous studies revealed that POSTN plays an important role in tumor development and is up-regulated in a wide variety of cancers [51]. A recent report noted that POSTN activated the Akt/PKB pathway via the αvβ3 integrin to promote cellular survival in colon cancer [52]. Similarly, Kudo et al. suggested that overexpression of POSTN may confer the ability to survive by inhibiting anoikis-related apoptotic pathways, thus allowing POSTN-overexpressing HNSCC cell line HSC2 to form colonies in soft agar and tumors in nude mice [53]. On the other side, Yan et al. utilized the siRNA gene knockdown approach in POSTN-expressing 293T cells to abrogate cell invasion in vitro and metastasis in vivo, which was proved to be associated with decreased cross-talk between integrinαvβ5 as well as EGFR pathways [54]. Taken together, increased expression of POSTN may be a common event of tumor development in laryngeal cancer and can be used as a useful marker to predict the cancer’s malignancy.

Above all, we identified several potential molecules to explain the mechanism of laryngeal cancer development. Taken together, overexpression of S100A2 significantly inhibited the invasion and migration capability of HEp-2 cells in vitro. It may provide useful targets for cancer gene therapy, which could allow transfer of the tumor-suppressor gene into tumor cells by viruses or other approaches without toxicity to surrounding normal cells, eventually achieving tumor eradication, preservation of laryngeal function, and successful treatment of laryngeal cancer.

## Supporting Information

S1 Table Patient demographic and clinical characteristics. (XLS)

S2 Table The list of proteins found to be expressed at different levels between laryngeal carcinoma samples and paired non-cancerous samples by iTRAQ analysis^a^. ^a^118:117 and 121:119 refer to relative level of protein expression in laryngeal carcinoma samples with respect to non-cancerous samples. Statistical calculation for iTRAQ-based detection and relative quantification were calculated using the Paragon Algorithm in the ProteinPilot software.
(XLS)

S3 Table The biological processes of differentially expressed proteins. (XLS)

S4 Table The protein classes of differentially expressed proteins. (XLS)

S5 Table The molecular functions of differentially expressed proteins. (XLS)
